# Integrated analysis of histone lysine lactylation (Kla)-specific genes suggests that NR6A1, OSBP2 and UNC119B are novel therapeutic targets for hepatocellular carcinoma

**DOI:** 10.1038/s41598-023-46057-4

**Published:** 2023-10-30

**Authors:** Qinjuan Wu, Xin Li, Menghong Long, Xianfeng Xie, Qing Liu

**Affiliations:** 1 Department of Anesthesiology, Chengdu Second People’s Hospital, Chengdu, China; 2 Department of Pain, The Affiliated Traditional Chinese Medicine Hospital of Southwest Medical University, Luzhou, China; 3 Department of Anesthesiology, The Affiliated Hospital, Southwest Medical University, Luzhou, China; 4 Department of Anesthesiology, The Affiliated Traditional Chinese Medicine Hospital of Southwest Medical University, Luzhou, China; 5 Hejiang Traditional Chinese Medicine Hospital, Luzhou, China

**Keywords:** Immunology, Biomarkers

## Abstract

Histone lysine lactylation (Kla) plays a vital role in the tumorigenesis of hepatocellular carcinoma (HCC). Hence, we focused on Kla-specific genes to select novel therapeutic targets. Differentially expressed Kla-specific genes (DEKlaGs) were identified from TCGA with the cut-off criteria |log_2_(FlodChange (FC))| > 2, p-value < 0.05, following investigating the prognostic value. The correlation between lactate accumulation and prognostic DEKlaGs expression was further investigated. On the other hand, we explored the roles of Kla activation in the immune microenvironment, immunotherapy, and drug resistance. We conducted gene set enrichment analysis (GSEA) to predict the pathways influenced by Kla. The predictive power of Cox model was further identified in ICGC and GEO databases. A total of 129 DEKlaGs were identified, and 32 molecules might be potential prognostic biomarkers. A Cox model including *ARHGEF37*, *MTFR2*, *NR6A1*, *NT5DC2*, *OSBP2*, *RNASEH2A*, *SFN*, and *UNC119B* was constructed, which suggested unfavorable overall survival in high-risk score group, and risk score could serve as an indicator for large tumor size, poor pathological grade and advanced stage. *NR6A1*, *OSBP2* and *UNC119B* could inhibit NK cell as well as TIL cell infiltration, and impair Type-I and II IFN responses in HCC, thereby contributing to unsatisfactory prognosis and immunotherapy resistance. *OSBP2* and *UNC119B* were identified to be related to chemotherapy resistance. GSEA showed that WNT, MTOR, MAPK and NOTCH signaling pathways were activated, indicating that these pathways might play a crucial role during the Kla process. On the other hand, we showed that *NR6A1* and *OSBP2* were overexpressed in GEO. *OSBP2* and *UNC119B* contributed to poor survival and advanced stage in ICGC. In summary, histone Kla was related to HCC prognosis and might serve as an independent biomarker. *NR6A1*, *OSBP2* and *UNC119B* were associated with the prognosis, immunotherapy, and chemotherapy resistance, suggesting that *NR6A1*, *OSBP2* and *UNC119B* might be novel candidate therapeutic targets for HCC.

## Introduction

Liver cancer is the sixth most common malignant tumor worldwide and the third leading cause of cancer-associated death^1^. Chronic liver diseases are the main causes that contribute to hepatocarcinogenesis, and risk factors include viral infection, such as hepatitis B virus (HBV) and hepatitis C virus (HCV), alcohol consumption, non-alcoholic fatty liver disease, and gene mutation. As the most prevalent pathological type^2^, the prognosis of hepatocellular carcinoma (HCC) is unsatisfactory, and the 5-year overall survival rate is less than 12% for advanced cases^2^. HCC is characterized by asymptomatic progression at the early stage and metastatic potential at the advanced stage, which leads to diagnosis at terminal stage and poor prognosis^3^. In recent decades, although therapy strategies for HCC, such as chemotherapy^4^ and immunotherapy^5^, have made great progress, overall survival has not improved^6^. Therefore, molecular and cellular events in HCC should be further understood to explore more effective therapeutic targets against HCC.

Histone lysine lactylation (Kla) is a newly identified posttranslational modification that can induce gene expression from chromatin directly^7^. Lactic acid serves as a metabolic fuel for cancer cells and contributes to the cell fate decision-making process^8^. Generally, lactic acid, the switch of Warburg effect or glycolysis, is abundantly accumulated in tumor microenvironment (TME), and it is the most crucial metabolic hallmark for malignant cells^9,10^. Histone Kla is sensitive to lactate levels. Inhibition of glycolysis impairs lactate production and then reduces histone Kla, while elevation of lactic acid generation can enhance Kla^7^. Lactate not only suppresses the recruitment of CTLs into the TME^11^ and induces the apoptosis of natural killer (NK) cells^12^, but also remodels macrophages and T cells to the immunosuppressive phenotypes, which contribute tumor cells to escape immune surveillance^13^. In addition, histone Kla may enhance genome instability, cancer growth, tumor angiogenesis, metastasis, and oncotherapy resistance^14^. In HCC, Jin et al. indicated that delactylation of *cyclin E2* induced by *SIRT3* could prevent cancer cell growth^15^. Lactylome analysis also showed that Kla preferentially influenced metabolic pathway-related enzymes, which play a vital role in regulating cellular metabolism and then promote HCC progression^16^. In addition, impairment of H3 histone lactylation by demethylzeylasteral (DML) may inhibit the tumorigenicity induced by liver cancer stem cells (LCSCs)^17^, suggesting that histone Kla is a potential candidate for the supplementary therapy of HCC. Therefore, we focused on Kla-specific genes to select potential therapeutic targets for HCC.

In our study, we obtained the Kla-specific genes as well as the gene expression profile of HCC from previous publications and The Cancer Genome Atlas (TCGA) respectively. Then, the differentially expressed Kla-specific genes (DEKlaGs) were identified by bioinformatics analysis. We screened out potential prognostic factors via univariate Cox regression, and then constructed a Cox model in line with prognostic DEKlaGs via multivariate and step-wise Cox regression. The role of lactate production in DEKlaGs expression was explored. We further determined the correlation between DEKlaGs expression and TME, immunotherapy, chemotherapy. We predicted the potential pathways influenced by Kla. Finally, the predictive power and prognostic value were identified in gene expression omnibus (GEO) and international cancer genome consortium (ICGC) databases.

## Materials and methods

### Data preparation and differentially expressed analysis

The HCC RNA expression profile and patients’ clinical information were downloaded from TCGA (https://portal.gdc.cancer.gov). A total of 50 normal controls and 374 tumor cases were obtained. Three tumor cases were excluded due to inadequate clinical information. In 2019, Zhao et al. identified the Kla-specific genes by ChIP-seq assay^7^. Furthermore, we downloaded Kla-specific gene lists (https://www.ncbi.nlm.nih.gov/pmc/articles/PMC6818755/#SD4) and then extracted their expression from the TCGA RNA expression profile. Subsequently, differentially expressed analysis was conducted in the R software limma package to select DEKlaGs with the cut-off standard |log_2_ (FoldChange (FC))| > 2 and p-value < 0.05.

### Identification of DEKlaGs prognostic value in HCC

According to DEKlaGs, univariate Cox regression analysis was used to select potential prognostic factors with the cut-off criteria of p-value < 0.05. Subsequently, we built a risk formula by multivariate and step-wise Cox regression analysis following prognostic DEKlaGs. We further calculated each patient’s risk score and divided them into high- and low-risk groups in line with the risk score median. Survival analysis along with long rank p-test was used to determine the overall survival between the two groups. Meanwhile, we conducted univariate and multivariate Cox regression analyses to explore whether the risk score was regarded as an independent prognostic biomarker. On the other hand, we analyzed the role of prognosis-associated DEKlaGs and risk score in HCC progression.

### Identification of lactate accumulation-associated genes in HCC

E1A binding protein p300 (*EP300*), hypoxia inducible factor 1 subunit alpha (*HIF1A*), lactate dehydrogenase A (*LDHA*) and B (*LDHB*) are associated with lactate production and accumulation in TME. Histone Kla is sensitive to lactate level. Zhao et al. demonstrated that these four genes played a crucial role during the Kla process^7^. Therefore, we investigated the expression and prognosis of these four genes in HCC. Meanwhile, we explored the correlation between these four genes and prognosis-associated DEKlaGs.

### Immune infiltration analysis

The relative infiltration levels of immune cell in each HCC patient was calculated through the single sample GSEA (ssGSEA) in line with genome expression in the R software^18^. Briefly, the markers of immune cells were obtained. The genome expression file was downloaded from the TCGA database. Then, the immunity file and gene expression profile were enrolled in R software simultaneously, and the immune cell scores and immune function scores were evaluated by gene expression level. In addition, the relative proportion of immune cells was determined by CIBERSORT (https://cibersort.stanford.edu/)^19^. Subsequently, we investigated the difference in immune infiltration between high- and low-risk group. Moreover, patients were further divided into high- and low-expression groups according to the median of each DEKlaGs expression. The difference in immune cell and immune function scores was investigated between the two groups. We further analyzed the correlation between prognosis-associated DEKlaGs and relative infiltration levels of immune cell.

### Immunotherapy analysis

The immune cell proportion score (IPS) about immunotherapy data for HCC was downloaded from TCIA (Table S1) (https://tcia.at/home). HCC patients were divided into high- and low-expression groups based on the median expression of each gene. The immunotherapy responses (ctla4-negative-pd1-neg, ctla4-negative-pd1-pos, ctla4-pos1-neg, and ctla4-pos1-pd1-pos) between the two groups were further analyzed. On the other hand, we investigated the co-expression between prognosis-associated DEKlaGs and immune checkpoints.

### Drug susceptibility analysis

Drug susceptibility information was obtained from the Cellminer database (Table S2) (https://discover.nci.nih.gov/cellminer/home.do). Generally, there were a total of sixty patients in the Cellminer database with gene expression profiles and drug susceptibility data. Hence, we evaluated the correlation between gene expression and drug susceptibility to explore the role of DEKlaGs in chemotherapy.

### Gene set enrichment analysis (GSEA)

To evaluate the signaling pathways influenced by Kla, we carried out gene set enrichment analysis (GSEA). In short, patients were divided into “high (H)” and “low (L)” groups in line with median expression. Furthermore, group files and gene expression profiles were imported into the GSEA_4.1.0 software. The gene-set database chose KEGG with the L group as the control group. Finally, the top three pathways influenced by Kla were listed.

### Identification of prognosis-associated DEKlaGs

To verify the prognostic value of the Cox model and prognosis-associated DEKlaGs, we downloaded the expression profiles of HCC from the GEO (https://www.ncbi.nlm.nih.gov/) and ICGC databases (https://dcc.icgc.org/). Briefly, GSE25097 and GSE54236 were obtained from the GEO database, and prognosis-associated DEKlaGs expression was extracted following differentially expressed analysis. On the other hand, the genes enrolled in Cox model were selected from ICGC, and then we calculated the risk score according to the established Cox model. We identified the predictive power of Cox model and prognostic value of DEKlaGs by survival and correlation analysis.

### Statistical analysis

SPSS (Version 23.3, IBM) software was used to difference statistics (p < 0.0001****; p < 0.001***; p < 0.01**; p < 0.05*).

## Results

### Histone Kla was an independent biomarker for HCC

**Figure 1:**
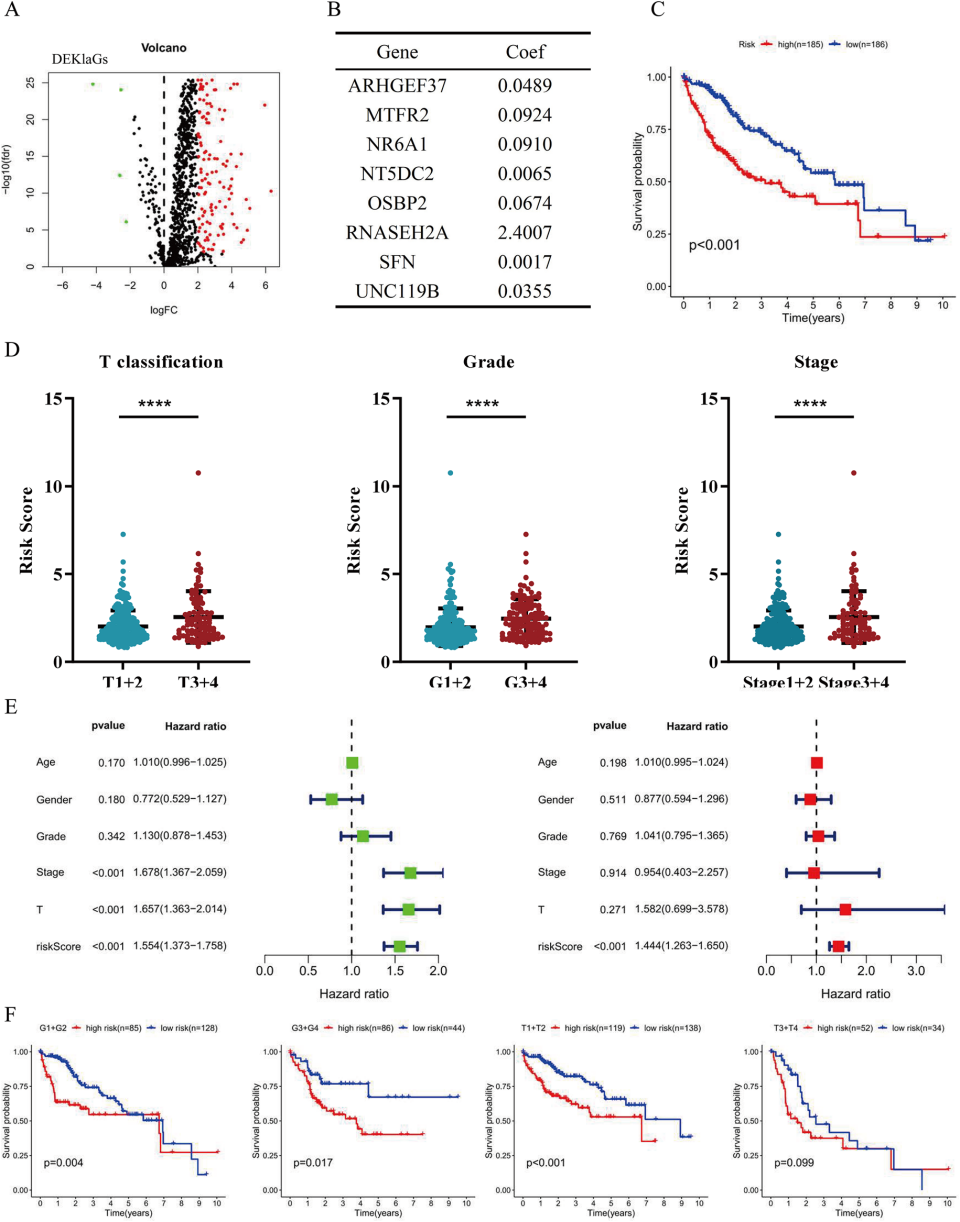
Histone lysine lactylation (Kla) was related to prognosis of HCC. (**A**) Volcano map of DEKlaGs in HCC with the cut-off criteria |log_2_FC| > 2, p-value < 0.05. (**B**) Cox regression model formula in line with DEKlaGs. (**C**) Survival analysis showed lower overall survival in high-risk score group than in low-risk score group. (**D**) Risk score in accordance with DEKlaGs might be an Indicator for large tumor size, poor pathological grade, and advanced clinical stage. (**E**) Univariate (left) and multivariate (right) Cox regression analysis based on risk score and clinical information, suggesting that risk score might be an independent biomarker for HCC. (**F**) Survival analysis along with pathological grade and T-stage. *DEKlaGs* differentially expressed Kla-specific genes, *FC* Foldchange, *HCC* hepatocellular carcinoma, *T* T-stage in TNM system.

To explore the prognostic value of Kla, we investigated the role of DEKlaGs in HCC prognosis. A total of 129 DEKlaGs were identified with the cut-off criteria |log_2_FC|> 2, p-value < 0.05 (Fig. 1A, Table S3). Among them, 32 genes were identified to be associated with the prognosis of HCC (Table 1). Furthermore, a Cox regression model was constructed through multivariate and step-wise Cox regression analysis according to the 32 prognosis-associated genes (Fig. 1B), which showed that patients with high-risk scores had a lower overall survival rate (Fig. 1C). The genes enrolled in Cox model were related to the poor prognosis of HCC patients (Fig. S1). High-risk scores according to Kla-specific genes were associated with large tumor size, poor pathological grade and advanced stage (Fig. 1D). Risk score was further identified as an independent prognostic biomarker for HCC (Fig. 1E), suggesting that these eight genes played a crucial role in HCC prognosis. On the other hand, stratification analysis of pathological grade and T-stage also confirmed the predictive power of the risk score (Fig. 1F). Although there was no significance in the T3 + T4 group because of fewer sample sizes, the prognosis in the low-risk group was relatively better than that in the high-risk group. These results suggested that Kla played a crucial role in HCC prognosis, and might serve as a novel prognostic biomarker.

**Table 1:** Thirty-two candidate prognostic molecules.

Gene	HR	Cox p-value	Gene	HR	Cox p-value
ANKRD13B	1.1161	0.0000	LRRC1	1.0380	0.0037
ARHGEF37	1.0415	0.0001	MMP9	1.0017	0.0491
BBS1	9.9352	0.0079	MTFR2	1.1458	0.0000
BCL9	1.0237	0.0004	NR6A1	1.1031	0.0000
CELSR3	1.1067	0.0138	NT5DC2	1.0083	0.0000
CIB2	1.0350	0.0380	NUDT17	1.0843	0.0028
DAGLA	1.0847	0.0008	OSBP2	1.0928	0.0000
DNMT3B	1.0739	0.0004	PPFIA4	1.2629	0.0025
EGLN3	1.0104	0.0286	PTPRN	1.9874	0.0078
GBA	1.0071	0.0002	RNASEH2A	6.4619	0.0069
HDAC11	1.0209	0.0003	RNF157	1.0413	0.0100
IQCC	1.2265	0.0000	RUSC1	1.0329	0.0000
KCNN1	1.4285	0.0028	SCRIB	1.0089	0.0049
LAPTM4B	1.0013	0.0005	SFN	1.0012	0.0008
LIMK1	1.0140	0.0034	SSR2	1.0027	0.0112
LRP4	1.0455	0.0105	UNC119B	1.0510	0.0013

### Histone Kla was upregulated in HCC

**Figure 2:**
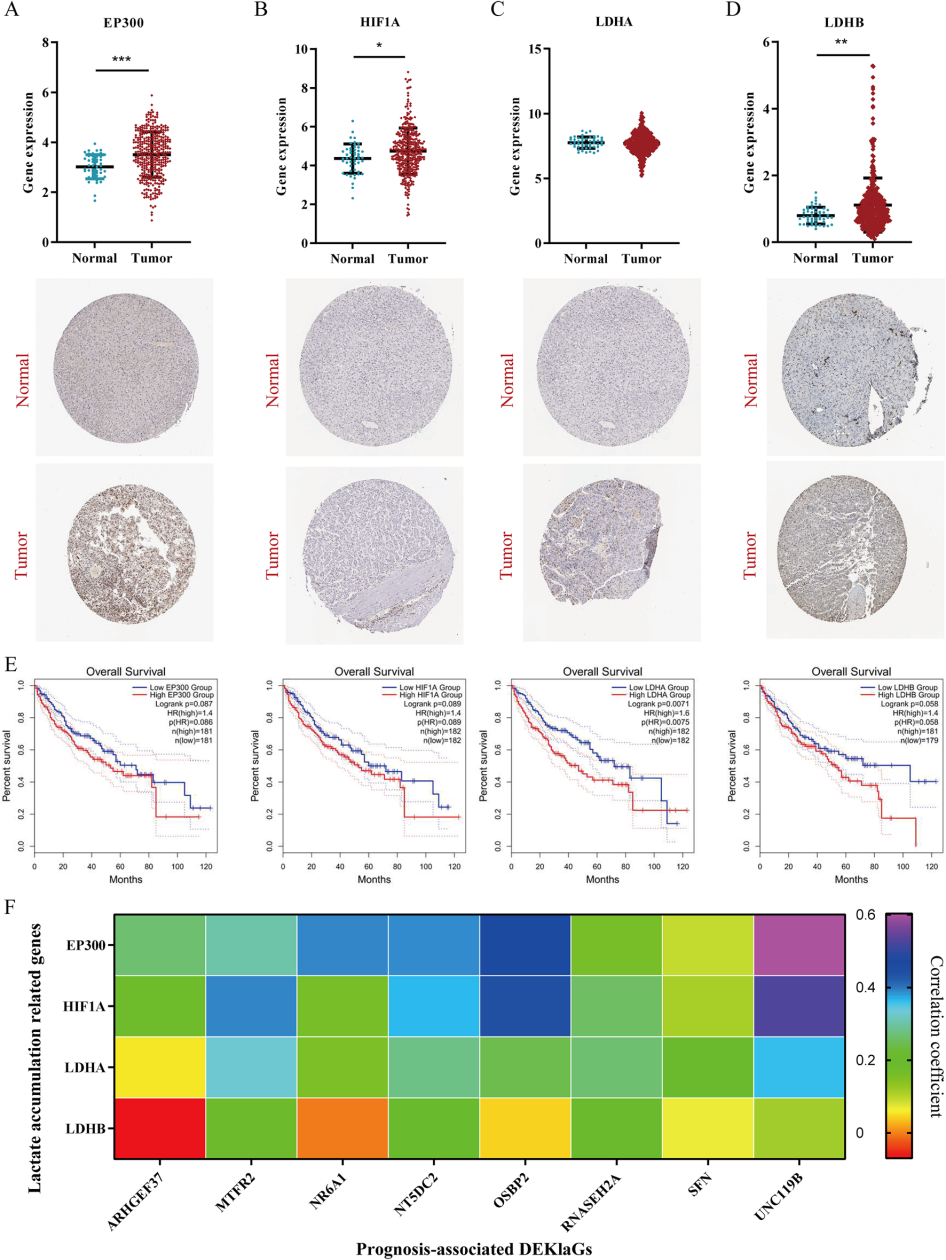
Kla-related genes were related to unfavorable prognosis. (**A–D**) Lactate accumulation-related genes including *EP300*, *HIF1A*, *LDHA*, and *LDHB* were overexpressed in HCC tissues. (**E**) High expression of Kla-related genes was associated with poor overall survival. Although there was no significance in survival analysis, patients with high expression of Kla activation-related genes tended to have an unsatisfactory prognosis. (**F**) Activation of Kla could induce prognosis-associated molecule expression.

To evaluate the total level of Kla in HCC, we analyzed the RNA and protein expression of Kla-related genes in HCC including *EP300*, *HIF1A*, *LDHA* and *LDHB*. As shown, lactate accumulation-associated genes were overexpressed in HCC tumor tissues (Fig. 2A–D). Patients with high lactate-accumulation tend to have poor overall survival (Fig. 2E). These results showed that high Kla level existed in HCC tumor tissues, which was related to an unsatisfactory overall survival rate. Furthermore, we identified that lactate accumulation-associated genes, especially *EP300, HIF1A* and *LDHA*, were positively related to prognosis-associated DEKlaGs expression (Fig. 2F), suggesting that these prognosis-associated DEKlaGs might be crucial candidate targets in HCC progression driven by Kla.

### Kla was associated with HCC immune infiltration

**Figure 3:**
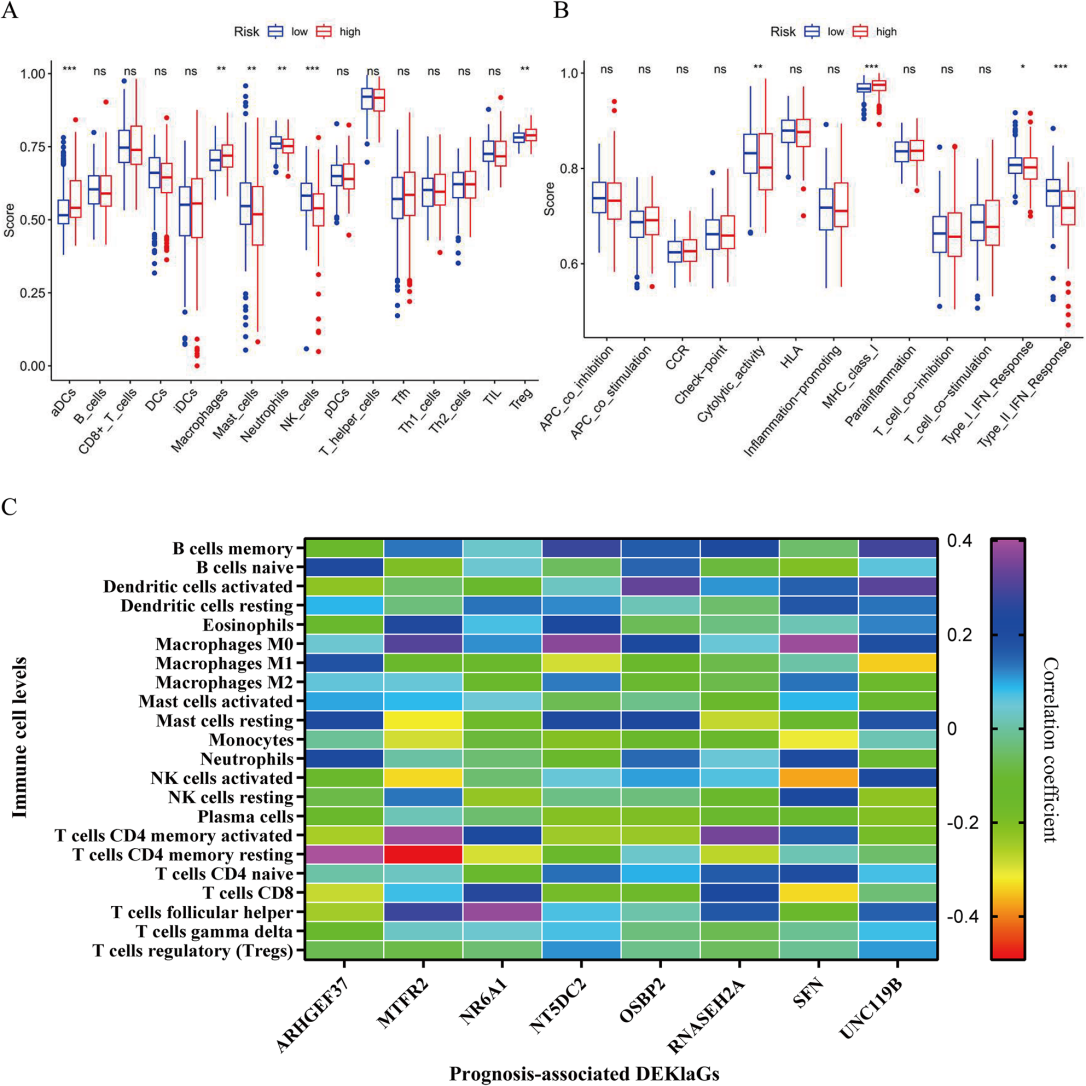
Kla was related to HCC immune infiltration. (**A**) High risk score was positively related to cancer progression-associated immune cells, especially T cells regulatory (Tregs). (**B**) Activation of Kla inhibited immune processes, including Type-I-IFN-Response and Type-II-IFN-Response. (**C**) Heatmap of the correlation between prognosis-associated DEKlaGs and infiltration level of each immune cell.

To explore the role of Kla in HCC TME, we analyzed the correlation between risk score and immune infiltration. As the results show, high-risk scores were related to lower immune cell scores, especially NK cell which plays a crucial role in inhibiting the proliferation and spread of tumor cells. On the other hand, T cell regulatory (Treg), as a vital part inhibiting immune responses and developing antigenic tolerance to tumor cells, was positively related to high-risk score (Fig. 3A). Meanwhile, the high-risk score was negatively related to immune response, especially Cytolytic-activity, type I and II IFN responses (Fig. 3B), thereby contributing to immune escape. High *NR6A1, OSBP2* and *UNC119B* expression resulted in low NK cell scores and impairment of IFN responses (Fig. S2A–H). Moreover, we explored the correlation between DEKlaGs and relative infiltration levels of immune cell. DEKlaGs expressions were positively related to Macrophage M2 infiltration while negatively related to NK cells (Fig. 3C, Table S4). These results suggested that Kla might inhibit the HCC immune process and play a crucial role in HCC immune infiltration.

### NR6A1, OSBP2 and UNC119B inhibit HCC immunotherapy

**Figure 4:**
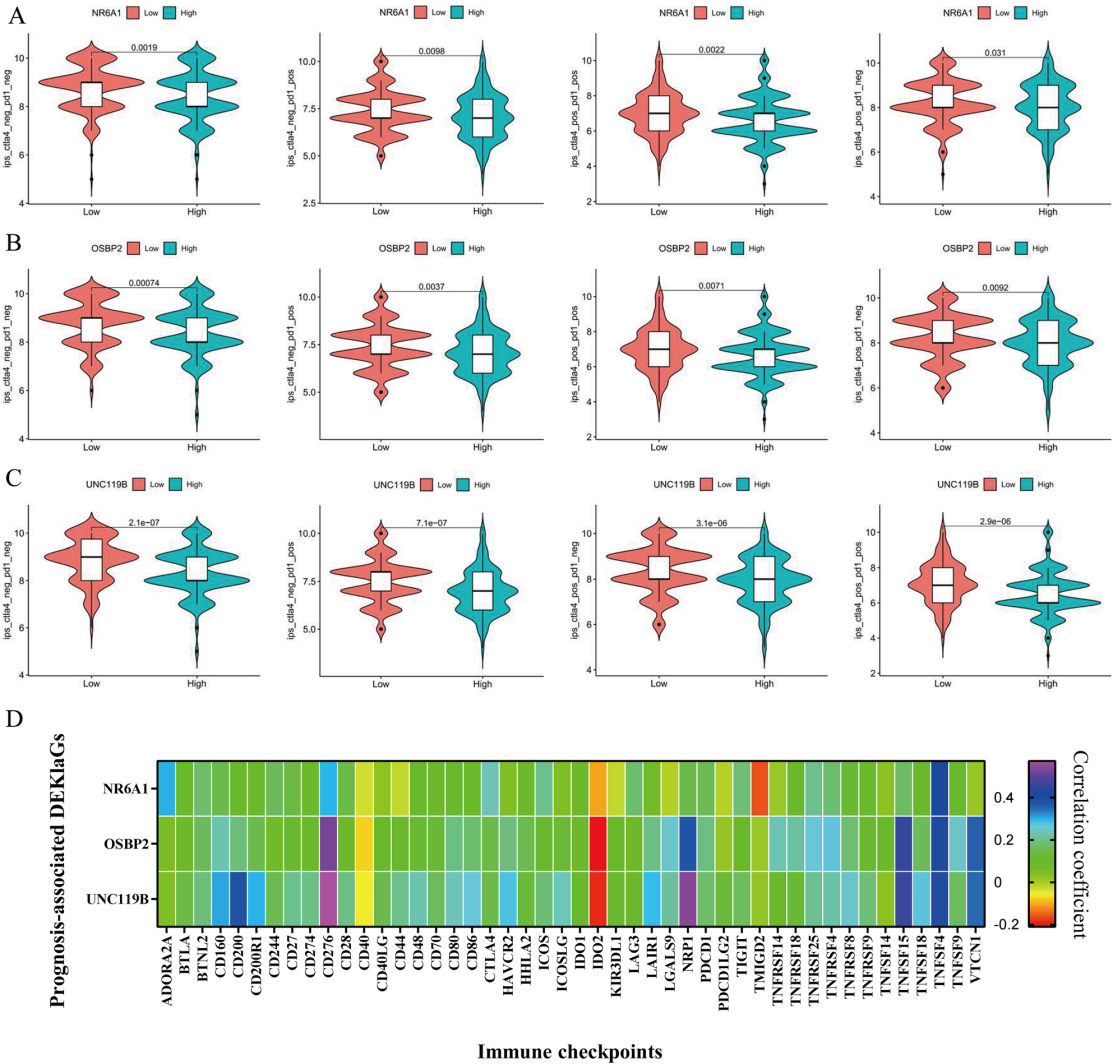
NR6A1, OSBP2 and UNC119B promoted immunotherapy resistance of HCC. (**A–C**) *NR6A1, OSBP2* and *UNC119B* were negatively associated with immune cell proportion score (IPS) including ips-ctla4-neg-pd1-neg, ips-ctla4-neg-pd1-pos, ips-ctla4-pos-pd1-neg, and ips-ctla4-pos-pd1-pos, suggesting that *NR6A1*, *OSBP2* and *UNC119B* contributed to immunotherapy resistance of HCC patients. (**D**) Expression of *NR6A1*, *OSBP2* and *UNC119B* was positively related to various immune checkpoints, such as *CD40*, *CD276*, *CALT4*, *TNFSF4*. These observations showed that *NR6A1*, *OSBP2* and *UNC119B* might be crucial immunotherapeutic targets for HCC.

We suggested that *NR6A1*, *OSBP2* and *UNC119B* inhibited NK cell function and immune function. We further explored their role in immunotherapy response. According to the IPS immunotherapy score from TCIA database, high *NR6A1*, *OSBP2* and *UNC119B* expression were all negatively related to four types of IPS scores, suggesting that the three targets played an inhibitory role in HCC immunotherapy (Fig. 4A–C). We further identified that *NR6A1, OSBP2* and *UNC119B* were positively related to majority of immune checkpoint expression, such as *CD28, CD276, CALT4, TNFSF4*, etc. (Fig. 4D). These findings demonstrated that *NR6A1, OSBP2* and *UNC119B* might play a critical role in HCC immunotherapy, suggesting that Kla might be regarded as a novel immunotherapeutic target for HCC.

### OSBP2 and UNC119B are related to HCC drug resistance

**Figure 5:**
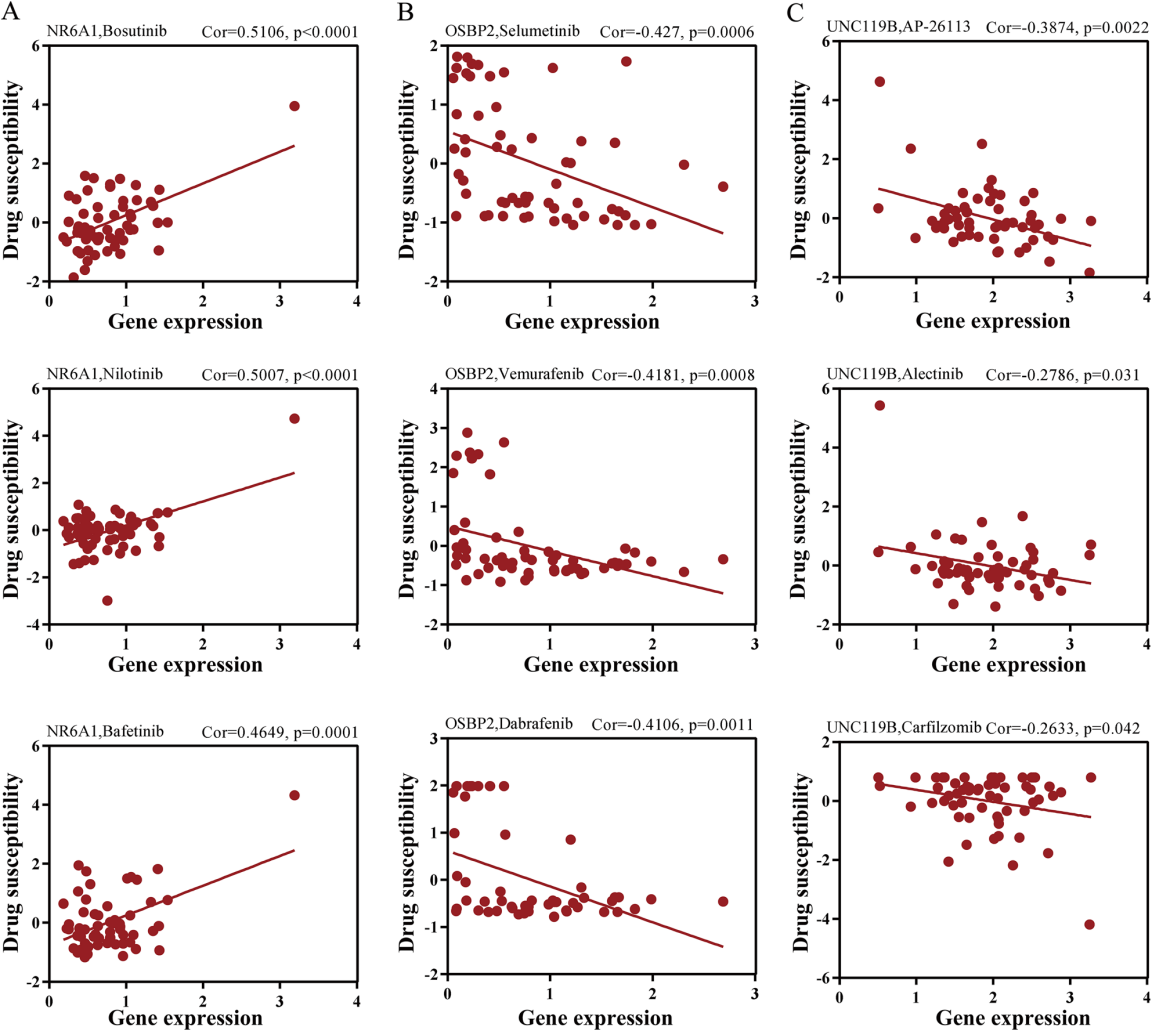
OSBP2 AND UNC119B enhanced chemotherapy resistance of HCC. (**A**) *NR6A1* was positively related to drug susceptibility. (**B**) *OSBP2* promoted Selumetinib, Vemurafenib, and Dabrafenib resistance of HCC patients. (**C**) *UNC119B* decreased the chemotherapy response to AP-26113, Alectinib and Carfilzomib.

To further investigate the role of Kla in HCC drug therapy, we obtained drug susceptibility data and conducted the correlation analysis. We found that *NR6A1*, as an oncogene, was positively related to drug susceptibility (Fig. 5A). The potential mechanism remains unclear and should be further explored. *OSBP2* decreased the susceptibility of the MEK1/2 inhibitor Selumetinib, the B-RAF inhibitor Vemurafenib and the Raf inhibitor Dabrafenib (Fig. 5B). *UNC119B* was related to chemotherapy resistance in HCC, such as Carfilzomib (Fig. 5C). These observations suggested that *OSBP2* and *UNC119B* inhibited the HCC immunotherapy response as well as susceptibility to chemotherapy, indicating that *OSBP2* and *UNC119B* might serve as crucial therapeutic targets for HCC.

### NR6A1, OSBP2 and UNC119B contribute to HCC-associated pathways activation

**Figure 6:**
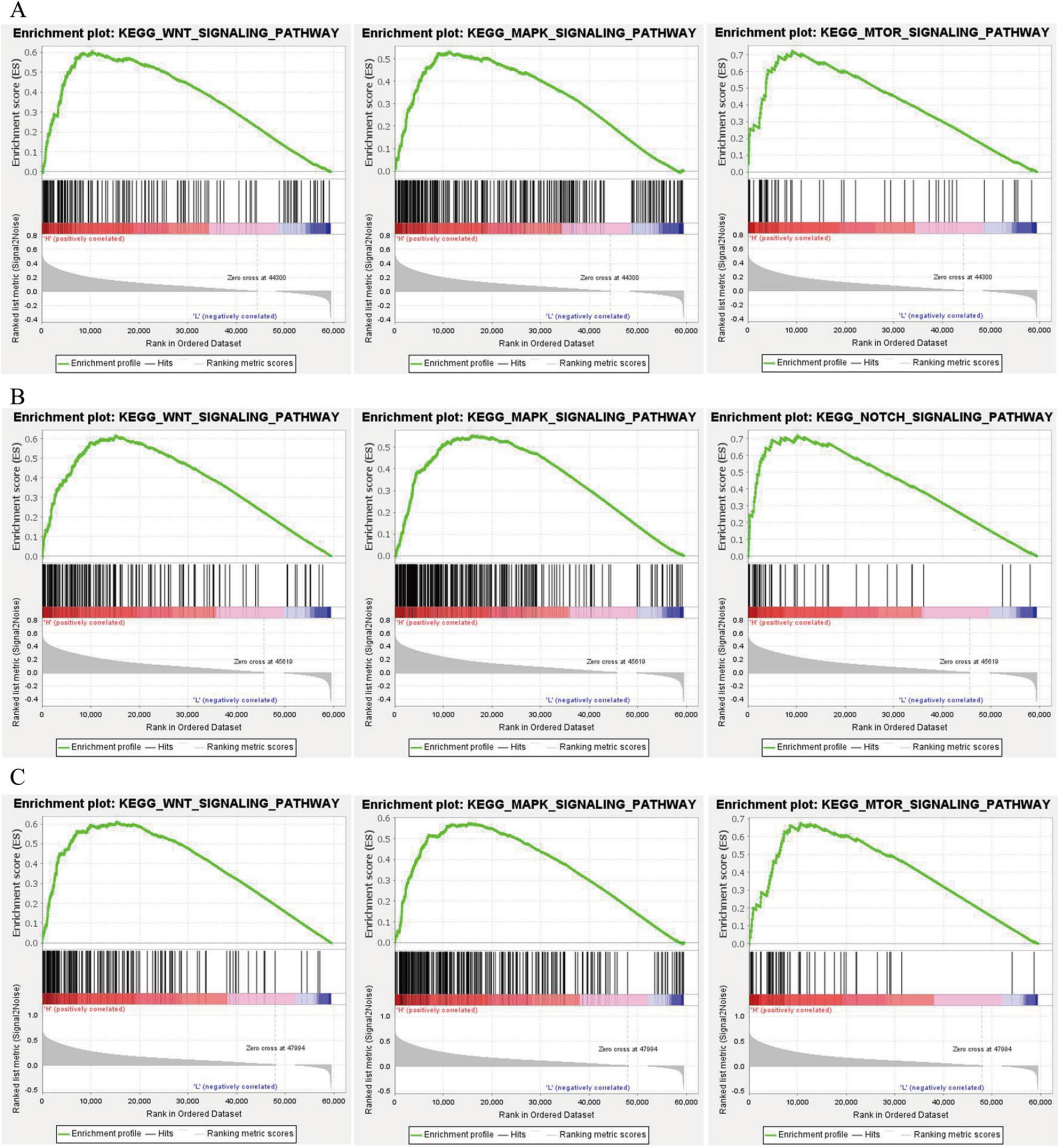
Kla induced activation of WNT, MAPK, NOTCH, MTOR signaling. (**A**) Overexpression of *NR6A1* was related to activation of WNT, MAPK and MTOR signaling pathways. (**B**) *OSBP2* induced the activation of WNT, MAPK and NOTCH. (**C**) Upregulated *UNC119B* promoted WNT, MAPK, MTOR signaling activity. The horizontal axis indicates a series of genes, while the vertical axis indicates the corresponding running ES. The peak of the curve is the ES. The black vertical lines represent the target genes in the gene set. The red meant bigger logFC, while blue is opposite. *ES* enrichment score.

To predict the potential mechanism, we carried out GSEA to select KEGG pathways influenced by *NR6A1*, *OSBP2* and *UNC119B*. As shown, *NR6A1*, *OSBP2* and *UNC119B* all contributed to the activation of the KEGG-WNT-signaling-pathway and the KEGG-MAPK-signaling-pathway, suggesting that these two pathways might be activated by histone Kla in HCC, and contribute to therapy resistance (Fig. 6A–C, Table 2). In addition, KEGG-MTOR-signaling-pathway was activated by *NR6A1* as well as *UNC119B*, and KEGG-NOTCH-signaling-pathway was activated by *OSBP2*. All of these signals were significantly relevant to HCC tumorigenesis, progression, metastasis, and therapy resistance, suggesting that targeting Kla might impair the activation of these pathways, thereby inhibiting HCC development and elevating the therapy response.

**Table 2:** Detailed information of GSEA.

Pathways	NES	Nominal p-value	FDR q-value
NR6A1			
KEGG_WNT_SIGNALING_PATHWAY	1.8906	< 0.0001	0.0085
KEGG_MAPK_SIGNALING_PATHWAY	1.7648	< 0.0001	0.0180
KEGG_MAPK_SIGNALING_PATHWAY	2.0201	< 0.0001	0.0017
OSBP2			
KEGG_WNT_SIGNALING_PATHWAY	1.9167	< 0.0001	0.0195
KEGG_MAPK_SIGNALING_PATHWAY	1.8141	< 0.0001	0.0136
KEGG_NOTCH_SIGNALING_PATHWAY	1.9300	0.0253	0.0215
UNC119B			
KEGG_WNT_SIGNALING_PATHWAY	1.8933	< 0.0001	0.0056
KEGG_MAPK_SIGNALING_PATHWAY	1.9058	0.0019	0.0046
KEGG_MTOR_SIGNALING_PATHWAY	1.9141	0.0019	0.0045

*NES* normalized enrichment score.

### Identification of NR6A1, OSBP2 and UNC119B in GEO and ICGC

**Figure 7:**
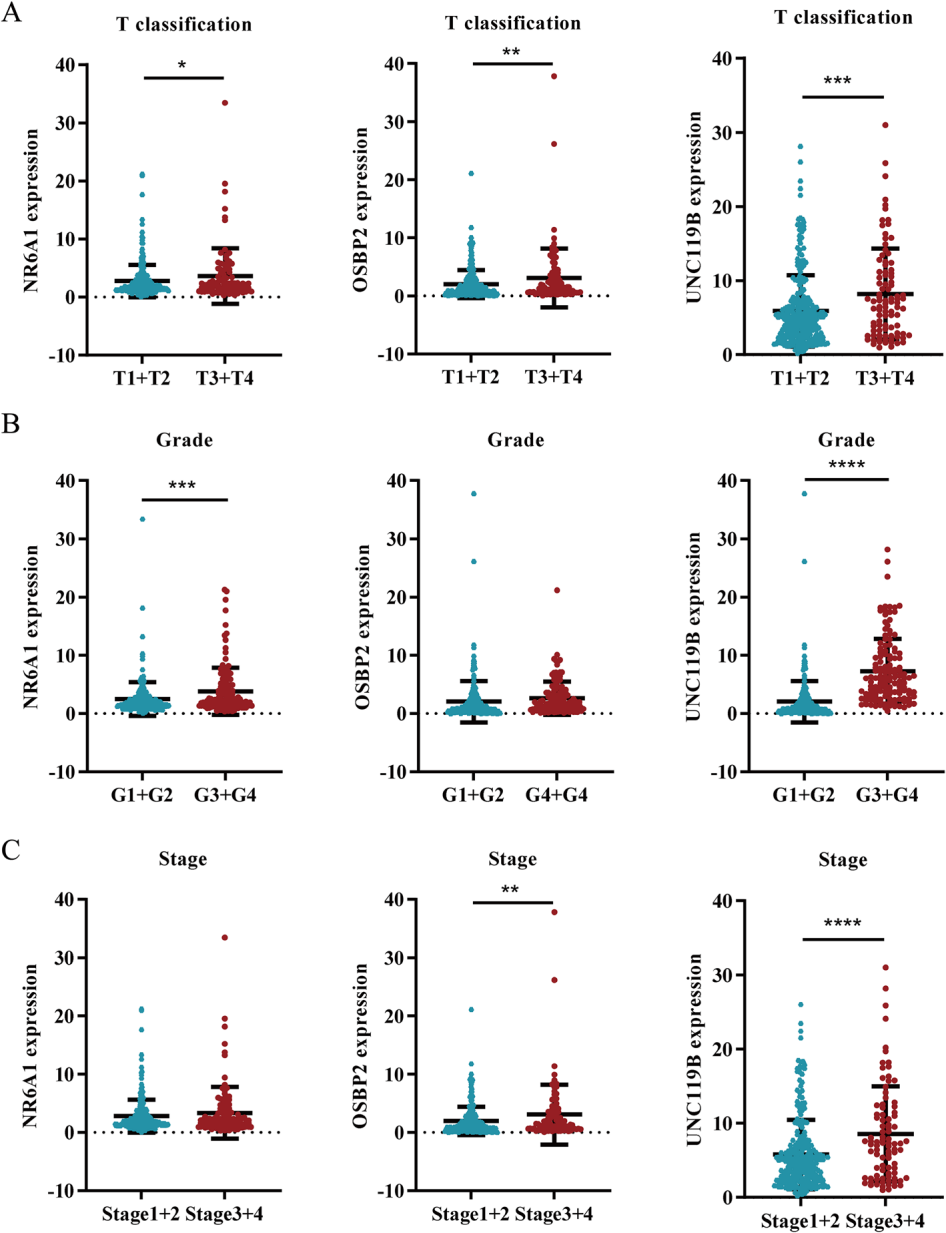
NR6A1, OSBP2 and UNC119B promoted HCC progression. (**A**) High *NR6A1, OSBP2* and *UNC119B* expression contributed to HCC proliferation. (**B**) *NR6A1* and *UNC119B* were related to the poor pathological grade of HCC. (**C**) *OSBP2* and *UNC119B* might be two advanced-stage biomarkers for HCC. These observations indicating that *NR6A1*, *OSBP2* and *UNC119B* played a crucial role in HCC progression, which shows promise to be three therapeutic targets.

**Figure 8:**
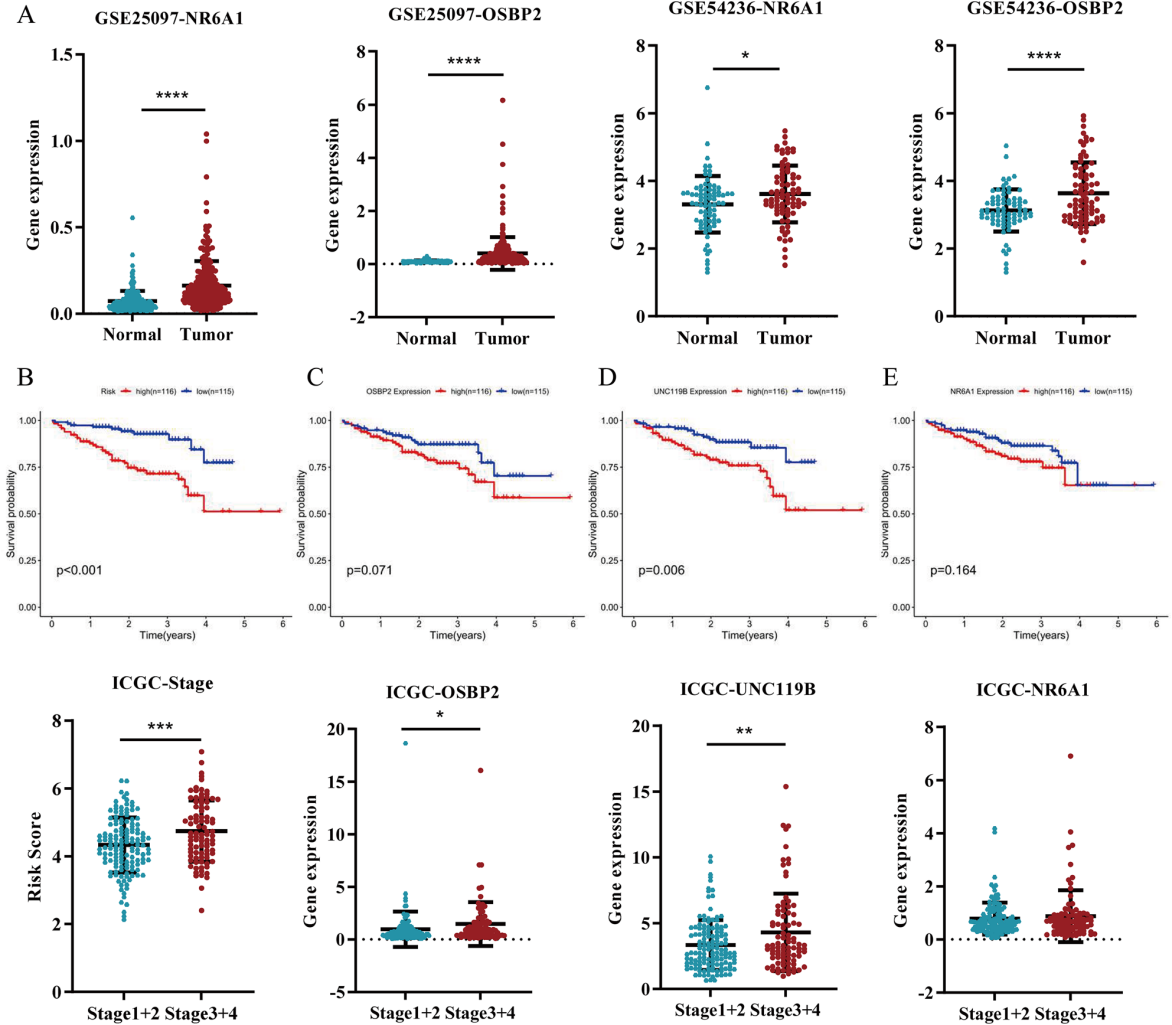
Identification of prognosis-associated DEKlaGs and Cox model in GEO and ICGC. (**A**) *NR6A1* and *OSBP2* expression in GSE25097 and GSE54236. *UNC119B* failed to search from GEO series, which showed that *NR6A1* and *OSBP2* were overexpressed in HCC cases. (**B**) Identification of Cox model in ICGC by survival and stage correlation analysis, suggesting that the risk formular according to prognosis-associated DEKlaGs could predict the prognosis of HCC patients accurately. (**C,D**) *OSBP2* and *UNC119B* were related to poor survival and advanced stage in ICGC. (**E**) NR6A1 showed no difference in ICGC database. These findings indicated that *OSBP2* and *UNC119B* might serve as two potential prognostic biomarkers for HCC.

To verify the role of *NR6A1*, *OSBP2* and *UNC119B* in HCC progression, we analyzed the correlation between gene expression and HCC tumor size, pathological grade and clinical stage according to TCGA database. *NR6A1, OSBP2* and *UNC119B* were associated with accelerated proliferation of HCC (Fig. 7A). *NR6A1* and *UNC119B* resulted in malignant pathological grade of HCC (Fig. 7B). High expression of *OSBP2* and *UNC119B* was related to advanced tumor stage of HCC (Fig. 7C). *UNC119B* was identified to play a carcinogenic role in tumor proliferation, pathological grade and stage, suggesting that *UNC119B* showed promise to be a crucial therapeutic target against HCC. Subsequently, we validated the gene expression in the GEO database, which indicated that *NR6A1* and *OSBP2* were overexpressed in HCC tissues (Fig. 8A). Nevertheless, *UNC119B* was not detected in GEO series. We further tested the prognostic value of DEKlaGs and predictive power of risk model in ICGC database. Risk model constructed in line with TCGA database could predict the overall survival and tumor stage of the samples in ICGC (Fig. 8B), suggesting that risk score might be an effective biomarker for HCC prognosis. On the other hand, *OSBP2* and *UNC119B* were also related to poor survival and advanced clinical stage in ICGC (Fig. 8C,D), while *NR6A1* showed no significance in ICGC (Fig. 8E). These findings suggest that *OSBP2* and *UNC119B* are expected to be novel therapeutic targets for HCC.

## Discussion

Cancer cells are characterized by reprogramming metabolic pathways, such as aerobic glycolysis, leading to lactic acid accumulation in the TME^20^. Lactic acid, as the major byproduct of aerobic glycolysis, contributes to cancer invasion and metastasis^20^. Moreover, lactic acid accumulation inhibits the immune process^21^, and leads to tumor immune escape via impairing tumor surveillance by T or NK cells^11^. Remodeling glycolysis is a common phenomenon in HCC, which is associated with HCC progression^22^, lenvatinib^23^ and chemoresistance^24^. Lactic acid accumulates in HCC TME during aerobic glycolysis, and histone Kla is sensitive to lactate level. An increasing number of studies have elaborated on the crucial carcinogenic role of histone Kla in HCC^17^. Therefore, we focused on Kla-specific genes to investigate the prognostic value of Kla and thereby select novel therapeutic targets for HCC.

In our study, we showed that risk score in accordance with DEKlaGs might be a predictor of poor prognosis. Eight molecules were enrolled in the risk formular. Among them, three genes, including *NR6A1*, *OSBP2* and *UNC119B*, were related to poor responses to immunotherapy and chemotherapy. *NR6A1*, known as germ cell nuclear factor, is a member of the nuclear receptor superfamily of ligand activated transcription factors^25^. *NR6A1* could regulate transcription indirectly by competing with transcription activators and regulating miRNAs. We found that *NR6A1* was upregulated in HCC tumor tissues, and related to accelerated proliferation and poor differentiation grade. In addition, high *NR6A1* could inhibit the function of immune cells and impair the IFN response, which leads to immunotherapy resistance. Recently, *NR6A1* could serve as a biomarker of disease progression and aggressiveness for prostate cancer (PCa), which showed that increased *NR6A1* immunoreactivity was remarkably related to advanced T-stage and cancer cell growth^26^. In HCC, *NR6A1* was identified as a prognostic marker, and associated with sorafenib resistance in HCC patients^27^. Lin et al. showed that *NR6A1* might inhibit the HCC immune response^28^ and activate the WNT signaling pathway as well as MTOR-signaling pathway^29^, which were similar to our study. On the other hand, NR6A1 could enhance HCC proliferation and invasion in vitro and vivo^29^, which indicated that *NR6A1* showed promise as a novel immune therapeutic target for HCC. *OSBP2* was first served as a cytosolic protein^30^, and had a high affinity for oxysterols^31^. Fournier et al. have shown that *OSBP2* is related to metastasis in breast and lung cancer, and identified *OSBP2* as a biomarker for tumor dissemination^32^. Recently, Shigemura et al. indicated that the *OSBP2* mutation could be detected in leukaemia^33^. In pancreatic ductal adenocarcinoma (PDAC), overexpression of *OSBP2* was associated with short survival, enhanced tumor progression, and chemotherapy resistance to gemcitabine (GEM) and 5-fluorouracil (5-FU)^34^. We suggested that *OSBP2* was positively related to high T-stage and advanced clinical stage, while negatively related to infiltration level of immune cells, such as NK cells and TIL. *OSBP2* also inhibited the HCC Type-I and II IFN responses. Moreover, *OSBP2* decreased the immunotherapy efficacy and drug susceptibility of HCC, suggesting that *OSBP2* might be a crucial therapeutic target for HCC. *UNC119B* is mainly associated with cilium assembly and lipoprotein transport. Unfortunately, there are fewer studies to explore its role in tumorigenesis and progression. In the present study, *UNC119B* was identified to be associated with HCC proliferation, poor pathological grade, and advanced stage, suggesting that *UNC119B* played a crucial role during HCC progression. On the other hand, *UNC119B* could inhibit the function of immune cells associated with tumor suppression and IFN response, leading to immune escape and immunotherapy resistance. Patients with high *UNC119B* expression contributed to shorter overall survival and impaired chemotherapy responses. Therefore, *UNC119B* was expected to be a novel therapeutic target for HCC, and further exploration of *UNC119B* in HCC might reveal the mechanism of immunotherapy and chemotherapy resistance.

Disorders of signaling pathways play a critical role in HCC tumorigenesis, metastasis, and therapy resistance. We found that WNT^35^, MAPK, MTOR, and NOTCH signaling pathway^36^, which are all related to poor prognosis of liver cancer, were activated by *NR6A1*, *OSBP2* and *UNC119B*. Selvaggi et al. assumed that targeting the WNT/β-catenin pathway was important to identify therapeutic agents for HCC^37^. Moreover, Morita et al. suggested that a combination of β-catenin inhibitors and immune checkpoint inhibitors (ICIs) might exert synergistic immunotherapy effects^38^. MAPK family includes *ERK, JNK*, and *p38*. Among them, the ERK pathway plays a crucial role in promoting cancer cell proliferation, migration, survival, and tumor progression in HCC. MAPK inhibitors could block cell proliferation and promote programmed cell death^39^, suggesting that inhibition of MAPK signaling might impair the malignant progression of HCC. The MTOR signaling pathway played a crucial role in HCC metabolism and contributed to HCC therapy resistance^40^, while the NOTCH signaling pathway was related to inflammatory microenvironment^41^, and targeting to NOTCH signaling pathways showed therapeutic potential for HCC patients^42^. On the other hand, mutual effects between the NOTCH and WNT signaling pathways played a vital role in HCC proliferation induced by tumor-associated macrophages (TAMs). Moreover, the WNT, MAPK, MTOR, and NOTCH signaling pathways were all remarkably activated in cancer stem cells (CSCs) of HCC, which contributed to metastasis and poorly differentiated with the worse prognosis^43^. Therefore, targeting histone Kla might impair activation of HCC-related pathways and then improve the prognosis. These observations suggested that Kla was expected to be a novel therapeutic target for HCC.

## Conclusion

Histone Kla was related to the HCC immune microenvironment, which might be regarded as an independent biomarker. *NR6A1*, *OSBP2* and *UNC119B* were identified to promote HCC progression, and lead to therapy resistance. In addition, *NR6A1*, *OSBP2* and *UNC119B* might induce the activation of HCC progression-associated pathways including WNT, MAPK, MTOR, and NOTCH signaling pathways. These findings suggested that *NR6A1*, *OSBP2* and *UNC119B* might be novel therapeutic targets for HCC immunotherapy and chemotherapy.

### Supplementary Information

Supplementary Information.

## Data Availability

All of the data presented in this study could be obtained from corresponding author.
