# Supplementary material for: Integrated analysis of histone lysine lactylation (Kla)-specific genes suggests that NR6A1, OSBP2 and UNC119B are novel therapeutic targets for hepatocellular carcinoma
Source: Sci Rep. 2023 Oct 30;13:18642. doi: 10.1038/s41598-023-46057-4 (PMC10616101; doi:10.1038/s41598-023-46057-4)
Supplement: Supplementary file 1 — Supplementary Information. [file 41598_2023_46057_MOESM1_ESM.zip › Revised-Supplementary files/Supplementary-Figures.doc]

Figure S1

Figure S1 Survival analysis of prognosis-associated DEKlaGs.

Figure S2

Figure S2 Differentially expressed analysis of immune cells in line with prognosis-associated DEKlaGs. A. ARHGEF37 was negatively related to CD8+ T cells, Th1 cells, Th2 cells and TIL. ARHGEF37 inhibited Cytolytic activity, Inflammation−promoting, T cell co-inhibition, T cell co-stimulation. B. High MTFR2 was related to higher Tregs level, and lower Type-II-IFN-Response. C. NR6A1 promoted Tregs and inhibited TIL and NK cells infiltration. NR6A1 inhibited the immune processes including Cytolytic activity, Type-I-IFN-Response and Type-II-IFN-Response. D. NT5DC2 was positively related to Tregs and negatively related to NK cells, and contributed to inhibition of Cytolytic activity, Type-I-IFN-Response and Type-II-IFN-Response. E. OSBP2 was negatively related to TIL, NK cells, CD8+ T cells, and also led to impairment of Cytolytic activity, Type-I-IFN-Response and Type-II-IFN-Response. F. RNASEH2A was mainly associated with Tregs and Type-II-IFN-Response. G. SFN was related to TIL and Type-II-IFN-Response. H. High UNC119B expression inhibited Type-I-IFN-Response, and tended to lower NK cells and higher Tregs level.
