# Supplementary material for: Integrated analysis of histone lysine lactylation (Kla)-specific genes suggests that NR6A1, OSBP2 and UNC119B are novel therapeutic targets for hepatocellular carcinoma
Source: Sci Rep. 2023 Oct 30;13:18642. doi: 10.1038/s41598-023-46057-4 (PMC10616101; doi:10.1038/s41598-023-46057-4)
Supplement: Supplementary file 1 — Supplementary Information. [file 41598_2023_46057_MOESM1_ESM.zip › Revised-Supplementary files/Table S3.doc]

| **Table S3 Differentially expressed Kla-specific genes in HCC** | | | | | |
| --- | --- | --- | --- | --- | --- |
| **Gene** | **Log_2_FC** | **Gene** | **Log_2_FC** | **Gene** | **Log_2_FC** |
| ABLIM2 | 2.26 | GNG4 | 6.32 | PYCR1 | 3.40 |
| ADCY2 | 2.62 | HCN2 | 3.96 | RAB3D | 2.44 |
| AHRR | 2.72 | HDAC11 | 2.52 | RCOR2 | 3.46 |
| ANKRD13B | 2.62 | HSF4 | 2.55 | RHOF | 2.55 |
| APLP1 | 4.03 | HSPB1 | 2.35 | RNASEH2A | 3.84 |
| ARG2 | 2.05 | IL1RL1 | -2.62 | RNF157 | 3.35 |
| ARHGEF37 | 2.24 | INSL6 | 3.50 | RUNDC3A | 2.76 |
| AS3MT | 2.85 | IQCC | 2.30 | RUSC1 | 2.26 |
| ASIC1 | 4.27 | JAG2 | 2.53 | SCRIB | 2.11 |
| BBS1 | 2.01 | KCNN1 | 3.30 | SERPINE2 | 2.16 |
| BCL9 | 2.27 | KIF5A | 3.99 | SEZ6L2 | 4.13 |
| BIK | 2.71 | KIF5C | 2.07 | SFN | 4.56 |
| C1QTNF6 | 2.08 | KIF7 | 2.08 | SHISA4 | 2.19 |
| CACNA1B | 4.69 | KIFC2 | 2.80 | SLC45A4 | 2.84 |
| CACNB3 | 2.04 | LAPTM4B | 2.47 | SLC6A9 | 2.99 |
| CACNG8 | 2.40 | LIMK1 | 2.12 | SMARCD3 | 2.20 |
| CARNS1 | 2.01 | LRP4 | 2.61 | SPECC1 | 2.43 |
| CASP12 | 2.18 | LRRC1 | 3.14 | SPEF1 | 2.41 |
| CD34 | 2.84 | MARCO | -4.20 | SSC4D | 2.35 |
| CELSR3 | 4.33 | MDGA1 | 3.06 | SSR2 | 2.01 |
| CIB2 | 2.45 | MMP10 | 4.92 | SYP | 2.54 |
| COL4A1 | 2.59 | MMP9 | 2.82 | TAL2 | 3.50 |
| CREB3L1 | 3.34 | MTFR2 | 3.93 | TBC1D31 | 2.18 |
| CSF3 | -2.23 | NAT14 | 2.04 | TLR9 | 2.07 |
| CSPG4 | 3.05 | NAT8L | 4.30 | TMEM119 | 2.55 |
| DAGLA | 2.23 | NECTIN4 | 2.87 | TMEM201 | 2.17 |
| DIO2 | 5.07 | NMB | 2.97 | TNFAIP6 | 2.82 |
| DNAJB13 | 2.55 | NR6A1 | 2.11 | TNFSF15 | 2.80 |
| DNMT3B | 2.65 | NRCAM | 3.21 | TNNT1 | 4.57 |
| DUSP9 | 5.95 | NT5DC2 | 3.58 | TPBGL | 3.72 |
| EGLN3 | 2.43 | NUDT17 | 2.71 | TRIM7 | 3.45 |
| EIF5A2 | 2.27 | OIT3 | -2.54 | TTC13 | 2.21 |
| FABP4 | 3.29 | OLFML2B | 4.13 | TUBB3 | 3.43 |
| FAM222A | 2.56 | OSBP2 | 2.65 | TXNRD1 | 2.20 |
| FBXW10 | 4.86 | PABPC1L | 2.49 | UNC119B | 2.02 |
| FER1L5 | 3.65 | PDZK1IP1 | 3.52 | UNC5A | 4.00 |
| FHAD1 | 3.08 | PLPP7 | 2.29 | USP49 | 2.11 |
| FIGNL2 | 2.62 | PLPPR3 | 2.32 | USP54 | 2.05 |
| FNDC10 | 2.38 | PPFIA4 | 3.62 | VARS2 | 2.39 |
| GALR3 | 2.11 | PRKCG | 2.34 | VGF | 3.85 |
| GBA | 2.17 | PROCA1 | 2.34 | VSIG10L | 2.70 |
| GINS4 | 2.70 | PTP4A3 | 3.28 | VWF | 2.16 |
| GIPR | 2.94 | PTPRN | 3.53 | WNT6 | 3.94 |
